# Zebrafish behavior feature recognition using three-dimensional tracking and machine learning

**DOI:** 10.1038/s41598-021-92854-0

**Published:** 2021-06-29

**Authors:** Peng Yang, Hiro Takahashi, Masataka Murase, Motoyuki Itoh

**Affiliations:** 1 Graduate School of Pharmaceutical Science, Chiba University, Chiba, Japan; 2 Graduate School of Medical Sciences, Kanazawa University, Kanazawa, Japan; 3 Aichi Institute of Technology Technical College of Communications and Electronics, Aichi, Japan

**Keywords:** Cognitive neuroscience, Behavioural methods

## Abstract

In this work, we aim to construct a new behavior analysis method by using machine learning. We used two cameras to capture three-dimensional (3D) tracking data of zebrafish, which were analyzed using fuzzy adaptive resonance theory (FuzzyART), a type of machine learning algorithm, to identify specific behavioral features. The method was tested based on an experiment in which electric shocks were delivered to zebrafish and zebrafish swimming was tracked in 3D simultaneously to find electric shock-associated behaviors. By processing the obtained data with FuzzyART, we discovered that distinguishing behaviors were statistically linked to the electric shock based on the machine learning algorithm. Moreover, our system could accept user-supplied data for detection and quantitative analysis of the behavior features, such as the behavior features defined by the 3D tracking analysis above. This system could be applied to discover new distinct behavior features in mutant zebrafish and used for drug administration screening and cognitive ability tests of zebrafish in the future.

## Introduction

Animal models play an important role in the scientific investigation of brain mechanisms involved in cognition, learning, and other behavioral functions^1–6^. Animal behavioral studies require the administration of experiments, video recording of the experiments, and parameter quantity analysis of the videos. To date, research on behavioral studies has mostly focused on two-dimensional (2D) data from videos recorded using a single camera^7^. Recently, researchers have become more interested in behavioral neuroscience using zebrafish because the developmental processes of zebrafish can be continuously visualized^8^, many genetic mutants have become available, and the fish are easily bred in great numbers and develop rapidly. Zebrafish (*Danio rerio*) has become a significant model organism in biological and medical research^9–12^. Despite these considerable advantages, studies on zebrafish behavior are lacking due to low-dimensional behavior data utilization and poor behavior analysis methods. Adult zebrafish are robustly social animals similar to humans^13,14^ and exhibit complex three-dimensional (3D) swimming patterns as reported in recent studies^15^. In a 3D design study, MacRì et al. found that 2D views may lead to inaccurate measurements of swimming activity in zebrafish, thereby requiring a general reconsideration of scoring zebrafish behavior to incorporate a 3D approach^16^. Most research analyzing model animal behavior has focused on simple parameters. Early works in this area focused primarily on the distance moved, velocity, tuning angle, etc.^7,17–20^. Recent studies of simple animal behavior features have applied boundary criteria to predefined parameters to quantify the proposed behavioral states^21–23^. Therefore, three further requirements can be applied to behavior analyses of zebrafish: (i) a productive method is required to collect high-dimensional behavior analysis and avoid false positives, (ii) an improved method is required to analyze complex behavior features, and (iii) a more effective method is required to describe and evaluate the newly identified animal behavior features.

Machine learning has attracted considerable attention from behavior researchers^24–26^. FuzzyART (Fuzzy Adaptive Resonance Theory) is a machine learning method with analog inputs that was developed to learn from new events without forgetting previously learned information. This model has shown robustness to variations in intensity and the detection of signals mixed with noise in the environment^27,28^. Several studies have shown that FuzzyART can be applied for expression profile analysis and protein classification based on 3D structures^29,30^.

In this work, we introduced a zebrafish 3D behavior feature recognition system that uses machine learning (FuzzyART). The 3D swimming path was reconstructed by our video capture and analysis system, and high-dimensional behavior data were analyzed by our machine learning algorithm. Moreover, we also developed a useful approach for evaluating the preidentified behavior features in a new data set.

## Methods

### Zebrafish and housing

A total of 10 adult (3-months-old) wild-type zebrafish were measured and analyzed in this study, and each fish was Zebrafish were kept in individual tanks at 28 ± 1 and pH 7.0 with a 14–10 h light/dark photoperiod (0900–2300 light) from 1-week zebrafish larvae to adult experiment age. Experiments were conducted during the light cycle. All animal experiments were approved by the Institutional Animal Care and Use Committee at Chiba University (Nos. 1–170, 2–174), and performed in compliance with the guidelines and regulations from Chiba university and Science Council of Japan (http://www.scj.go.jp/ja/info/kohyo/pdf/kohyo-20-k16-2e.pdf) and the ARRIVE guidelines for involvement of animals (fish).

### Experimental setting and electric stimulus treatment protocol

The animal behavior data collection and electric stimulus treatment were automatically controlled using an Arduino Uno microcontroller^31^ (Arduino project’s foundation, Italy) by our custom program. A filled with 5L water cubic tank (20 cm length 20 cm width 20 cm height) was used to observe fish behavior, and a red light-emitting diode (LED) was placed on the outside of the tank. At the beginning of the experiment, the LED indicator was turned on so that ventral and lateral videos are synchronized by our custom MATLAB program. Low-intensity electrical stimulation was administered to the zebrafish to induce behavioral changes. Two stainless steel woven wire meshes (30 mesh Type 304, Kuho Metal Manufacturing Co., Ltd, Japan) were installed as electrode plates (15 V DC) on the left and right sides of the tank. The electric stimulus was also automatically controlled by the Arduino Uno. A flowchart was generated to illustrate the electric stimulus treatment protocol: QT phase: 30 s of quiescent time for free swimming; and ES phase: electric stimulus (500 ms on and 500 ms off).

### Video-tracking analysis

Video tracking was performed using an EthoVision XT10 (Noldus Information Technology) based on recorded videos, and the maximum sample rate was 60.0 frames per second (fps). A tracking analysis was configured to begin after the subject was detected for more than 1 s. Detection settings (grayscale method or dynamic background method) were selected to most accurately acquire zebrafish behavior. Movement tracks were smoothed (across ten samples) and examined for abnormalities (e.g., missing samples, reflection clustering, or rogue points) by EthoVision XT10. Then, the standard 2D swim track of zebrafish was generated. Next, exports and tracks were interpolated to replace missing values and exported into a CSV file.

### 3D swim path reconstructions

Animal behavior was recorded by two cameras (Sony AZ1 Action Camera, Japan), and the time lag between two videos (ventral and lateral) was synchronized by an LED indicator. At the beginning of the experiment, the LED indicator was automatically turned on by the Arduino microcontroller. The beginning frame of each video was detected via a computer vision analysis using a customized MATLAB program to ensure the accuracy of the spatiotemporal behavioral data. After time synchronization, 3D swimming path reconstruction was performed by using customized and R programs. The custom code will be shared freely for noncommercial use (Project Website: https://github.com/singularpse/Zebarafish_3D_swim_path_reconstructions_system).

### Clustering analysis

A FuzzyART model is a rapid stable machine learning algorithm that responds to arbitrary sequences of analog inputs^27^. An unsupervised FuzzyART was used to detect new behavior patterns linked to the treatment in our study. A binomial test was used to analyze the associations between the cluster and treatment (ES: electric stimulus). The probability of correlation was not significantly different from 0.5 for each of the clusters (two-tailed binomial test). A grid search technique was used to determine the optimal parameter values (cumulative proportion of variance explained and vigilance for each cluster) of the clustering algorithm in different time segments.

### Web application "ShinyR-3D-zebrafish"

To decrease the complexity and time required to visualize and analyze the data, we developed a new, free, open-source, cloud-based application that has an intuitive graphical user interface that enables novice users to perform complex analyses quickly. Model parameter selection includes the time segments (Time_block), cumulative proportion of variance explained (pca), vigilance for each cluster (vi), time when the behavior occurred (trackID), and number of behaviors shown in the plot. Moreover, users could also check informative data tables, 3D-tracking plots or animations, and behavior 3D plot summaries by this interactive web application "ShinyR-3D-zebrafish" (Fig. S1, see the demo page on our site: https://singularpse.shinyapps.io/review_raw_3d/).

### Experiment protocol

A total of 10 adult (3-months-old) wild-type zebrafish were used in this study and each zebrafish behavior was measured individually as described in “Experimental setting and electric stimulus treatment protocol”. The zebrafish was placed in the tank for 10 min before conducting experiments to adapt to the new environment. When the electrical stimulation protocol is executed, the dual camera system starts recording video at the same time. After the two angles of video are processed by post-synchronization, video tracking is performed, and all the exported behavior data is used as a data set for the machine learning system for analysis.

### Data analysis

Histograms are presented as the mean ± standard error of the mean (SEM). All the plots were generated by a program code developed in R version 3.6.1^32^. Statistical analyses were performed using two-tailed Student’s t-tests and Wilcoxon signed-rank tests based on codes developed in R version 3.6.1. For the data that met the assumption of normality and homogeneity of variance, a two-way ANOVA was conducted to compare the effectiveness and Tukey’s honestly significant difference tests was used for post hoc comparisons. The Shapiro–Wilk test for normality and Bartlett test for homogeneity of variances were carried out. Two-sample Kolmogorov–Smirnov tests were performed for two random samples from identical populations. In all comparisons, p < 0.05 was considered to indicate statistical significance. Programmed control of electric stimulus and light was performed using the Arduino board and open-source Arduino Software (IDE, version 1.8.10)^31^. An analysis of the video time synchronization was performed using MATLAB R2017b (MathWorks) with the Computer Vision Toolbox^33,34^. The 3D reconstruction and 3D spatiotemporal reconstruction of the swim path were performed based on a previously described method using the R packages plotly^35^, ggplot2^36^, gg3D. FuzzyART was used to perform clustering to link the treatment to the alteration of all behaviors. The R package ggplot2 was utilized to visualize the FuzzyART clustering results.

## Results

### Zebrafish 3D swimming path reconstruction using two-camera video data

**Figure 1:**
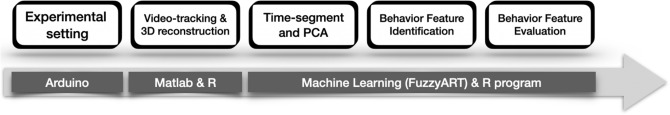
Flowchart illustrating the experimental strategy of this study. The experimental workflow included recorded novel tank test behaviors across treatments and trials. A video-tracking analysis was performed and 3D swimming path reconstruction was performed by using customized MATLAB and R software. Time-segmentation and dimensionality reduction of the 3D spatiotemporal data were performed by a principal component analysis (PCA), followed by unsupervised clustering by using customized machine learning algorithms (FuzzyART) across all behavioral spatiotemporal data to identify potential behavior features linked to treatment. Finally, a behavior feature evaluation was performed with the trained model and new input data.

**Figure 2:**
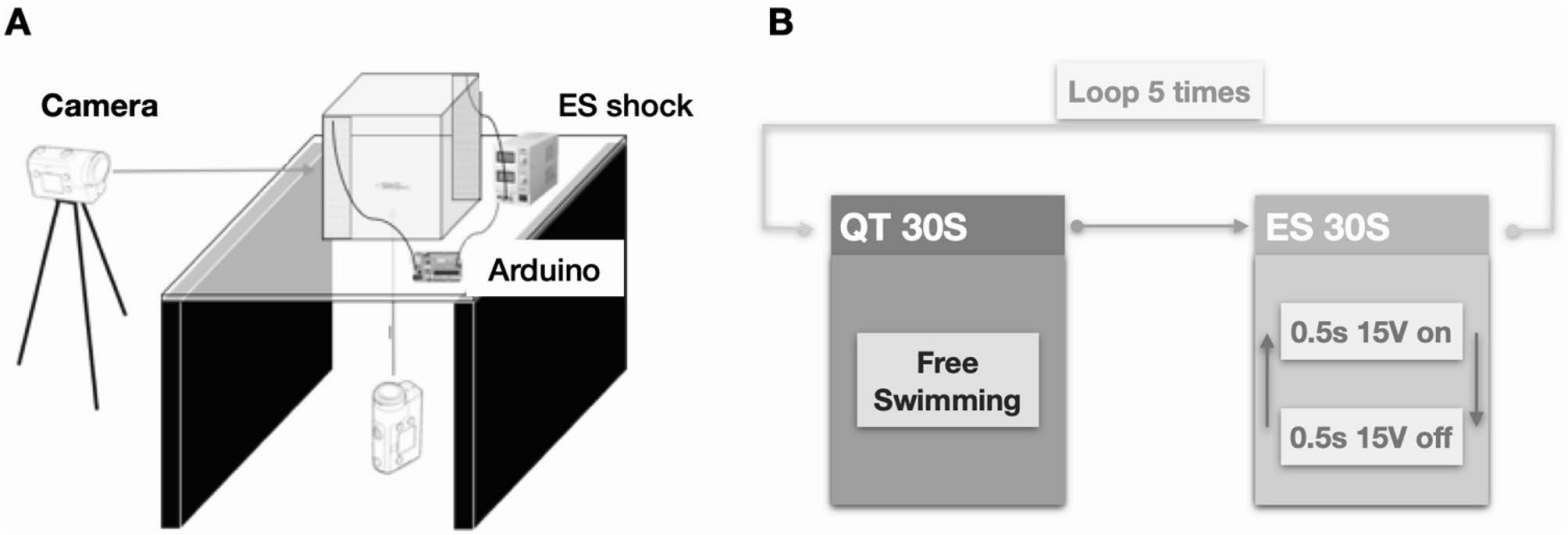
Schematics of the setup to acquire 3D trajectories in response to the experimental stimuli. (**A**) Experimental devices include an Arduino (open- source microcontroller)-based programmed electric stimulus system and a two-camera video capture system. Naive wild-type zebrafish were placed in an unfamiliar novel tank. Animal behavior was manually observed, and two cameras recorded videos for automated analysis in EthoVision XT10. Tracking data for each subject were exported, processed and visualized by customized MATLAB and R software programs. (**B**) Flowchart illustrating the electric stimulus treatment protocol: QT phase: 30 s of quiescent time for free swimming and ES phase: electric stimulus (500 ms on and 500 ms off) was delivered to the zebrafish.

The following experimental design was used: 1. experimental setting; 2. video tracking and 3D swimming path reconstruction; 3. 3D swimming path data time-segment and dimension reduction by principal components analysis (PCA); 4. machine learning model training and behavior feature identification with that data; and 5. behavior feature evaluation with the trained model and new input data (Fig. 1). The 3D swimming path reconstruction system includes an Arduino (open-source microcontroller)-based two-camera video capture system (Fig. 2A) and a programmed electric stimulus system (Fig. 2B). Naive wild-type zebrafish were placed in this system, and an electric stimulus was delivered to the zebrafish following the stimulus protocol: 30 s QT (quiescent time) phase for free swimming and 30 s ES (electric stimulus: 500 ms on and 500 ms off) phase. Animals were recorded by two cameras for automated analysis. Tracking data for each zebrafish were exported, processed, and visualized by customized MATLAB and R software programs.

**Figure 3:**
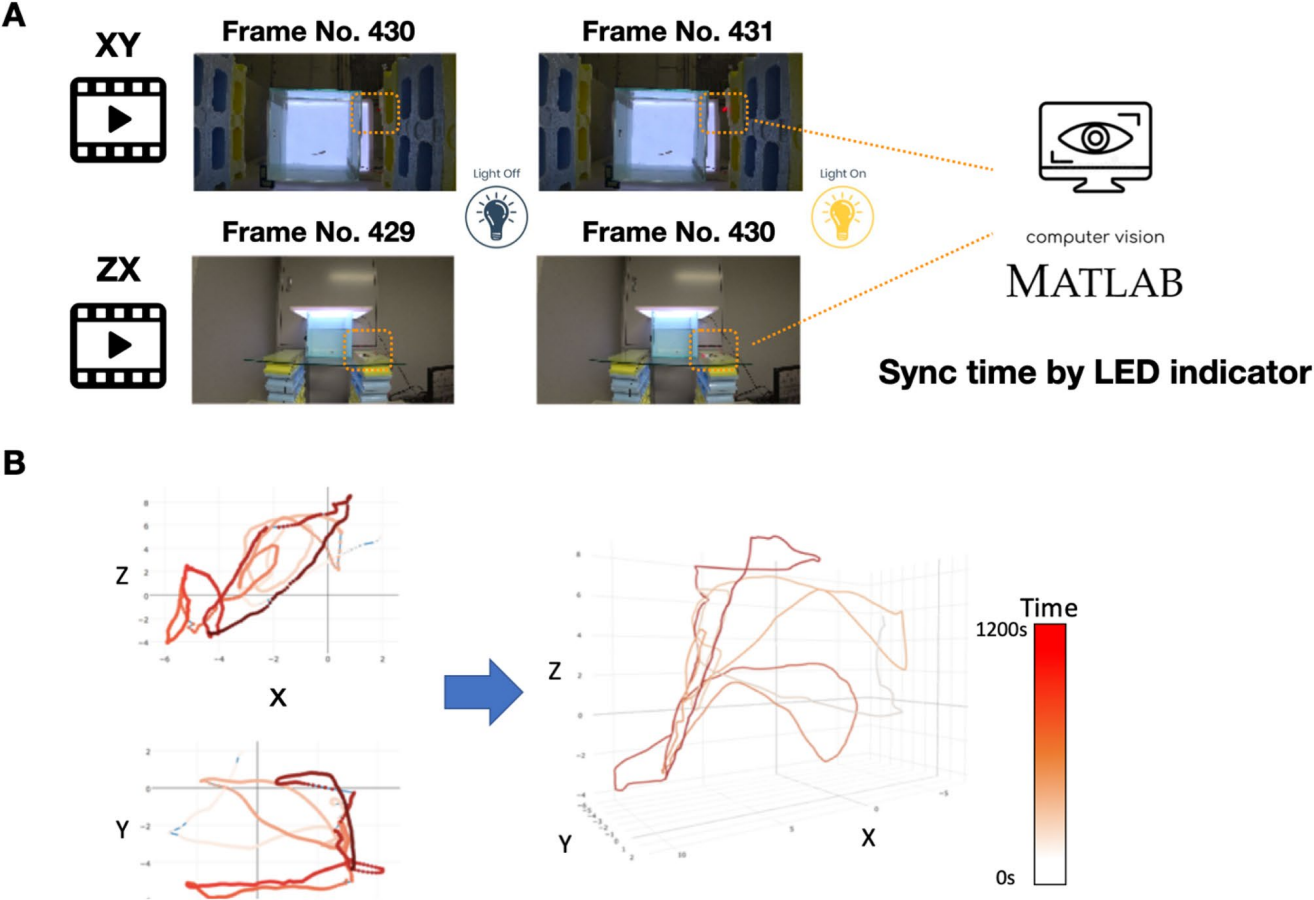
Camera time synchronization and swim path reconstructions in adult zebrafish. (**A**) Animal behavior was manually observed, two cameras recorded the videos, and the time lag between two videos (ventral and lateral) was synchronized by an LED indicator. At the beginning of the experiment, the LED indicator was automatically turned on by Arduino. The experiment’s beginning frame of each video was detected via a computer vision analysis by a customized MATLAB program to ensure the accuracy of behavioral spatiotemporal data. (**B**) After time synchronization, 3D swimming path reconstruction was performed using a customized R program.

To synchronize the two movies from the cameras while capturing 3D time-series behavior data of the zebrafish, we added an LED indicator controlled by a microcontroller, and the time lag between two videos (ventral and lateral) was eliminated. At the beginning of the experiment, the LED indicator was automatically turned on by the Arduino microcontroller. The LED-ON frame of each video was detected with computer vision analysis by a customized MATLAB program to ensure the accuracy of the behavioral spatiotemporal data (Fig. 3A). After time synchronization, 3D swimming path reconstruction was performed using a customized R program (Fig. 3B).

### Treatment (ES)-associated behavior features were identified by machine learning

**Figure 4:**
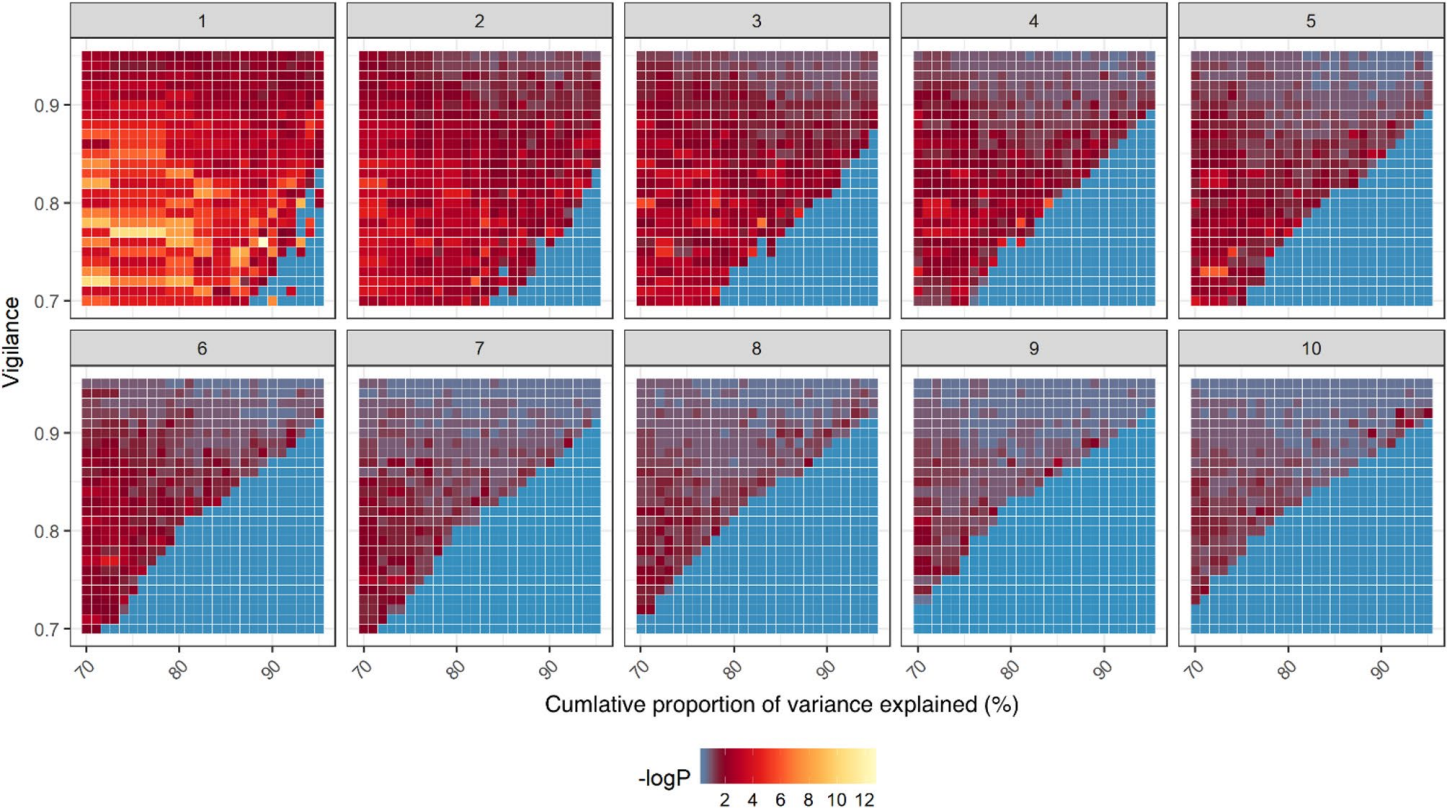
Clustering showed that a higher –log_10_ (*p*)) value will lead to ES-specific behavior. For the different time segment behaviors (1 ~ 10 s, figure panel label), the correlation between the cluster and treatment was shown in each tile under different clustering analysis conditions. A binomial test was performed to examine whether the behavior feature was significantly biased toward the treatment (ES). The legend is shown below the heatmap, with the minimal -log10 (p) of the binomial test of the top ES-specific behavior feature on that clustering analysis condition shown in dark blue and high –log10(p) shown in yellow.

**Figure 5:**
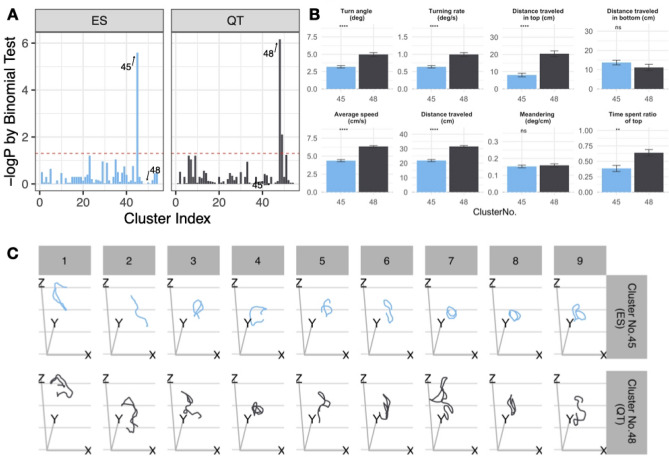
Behavioral features identified by machine learning. (**A**) Correlations between the cluster of behavior features and treatment of the optimal parameter values found by grid search (shown in Fig. 4) in 5 s time segment behavior. Cluster no. 45 was significantly associated with treatment (ES phase); cluster no. 48 was significantly associated with no treatment (QT phase). The cluster identifier number is shown on the x-axis, and -log p-values are indicated on the y-axis. The horizontal red dashed line shows a p-value of 0.05 by binomial test (the probability of correlation was not significantly different from 0.5 for each of the clusters). (**B**) Exploratory behavioral profiles of zebrafish between cluster no. 45 (ES phase) and cluster no. 48 (QT phase), which were newly identified by the clustering analysis. (**C**) Reconstruction and visualization of the 3D swim path of adult zebrafish between cluster no. 45 (ES phase) and cluster no. 48 (QT phase). Because of the limitation of the picture size, only 9 motion tracks in each behavior feature are displayed. Data are shown as the mean ± SEM for zebrafish (n = 10, Student's t-test), with p < 0.05 and p < 0.01 represented by * and **, respectively.

Next, we used machine learning to identify the behavioral features of adult zebrafish. An unsupervised clustering analysis via FuzzyART was used to detect new behavior features linked to the treatment in our study^27,37^. The clustering analysis included time-series data segment (1, 10 s), dimension reduction, clustering analysis by FuzzyART, treatment-specific analysis and results visualization. We used a grid search technique to determine the optimal parameter values (cumulative proportion of variance explained and vigilance for each cluster) of the clustering algorithm in different time segments (duration per occurrence of each behavior feature). As shown in Fig. 4, the binomial test was used to analyze the association between the cluster and treatment (ES), and the ratio of ES phase time in all experiments (50%) was used as the expected probability in the binomial test. The result of the correlation between the cluster and treatment under different clustering analysis conditions was visualized by a heatmap. Considering that the analysis of behavior features needs to balance the statistical significance and the length of observation time (although shorter time segments are prone to have significant differences, they may lose critical behavioral features as a trade-off), we chose a time segment of 5 s and selected the cluster analysis conditions that showed the highest statistical significance under this time segment for the next study (cumulative proportion of variance explained: 72%; and vigilance for each cluster: 0.73). We focused on the newly identified behavior features by machine learning in 5 s time segments. As shown in Fig. 5A and Fig. S2, cluster no. 45 included newly identified behavior features that were significantly associated with the treatment (ES) and cluster no. 48 was significantly associated with no treatment (QT).

To validate the behavioral features identified by machine learning, we compared the results with traditional manually quantified behavioral parameters in cluster no. 45 in ES and those in cluster no. 48 in QT. A significant difference was observed in the turn angle, turning rate, distance traveled in top, average speed, distance traveled, and time spent ratio of top compared with the cluster no. 48 behavioral features (Fig. 5B). Next, we developed an open-source cloud-based application (ShinyR-3D-zebrafish) to visualize the 3D behavior of animals. A 3D snapshot of adult zebrafish and a video of cluster 45 no.1, 3, and 5 were shown in Fig. 5C and Supplementary Video S1, respectively. The results showed that the movement distance of cluster no. 48 was shorter than that of cluster no. 45. In addition, users could adjust the watching angle and obtain more information about these newly identified behavior features (Fig. S1, see the demo page on our site: https://singularpse.shinyapps.io/review_raw_3d/).

### Evaluation of the preidentified behavior features in the new data set by machine learning

**Figure 6:**
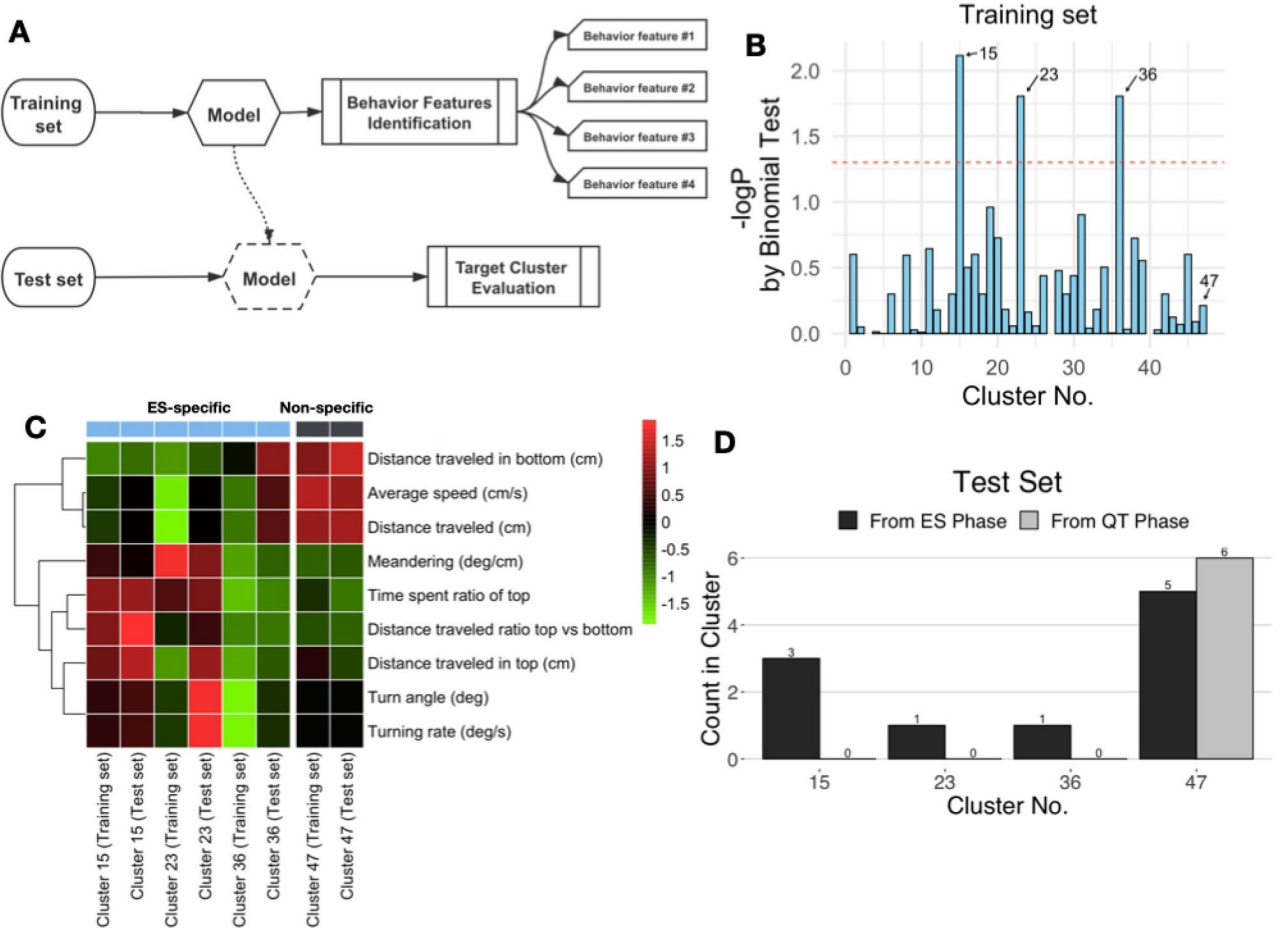
Evaluation of the behavior features newly identified by machine learning. (**A**) Fit model was trained on the training set data, and the new treatment-specific behavior feature was identified. The test set was used to evaluate the behavior feature newly defined on the training set. (**B**) ES-specific behavior feature screening in the training set. Cluster nos. 15, 23, and 36 were significantly associated with the treatment (ES-specific); cluster no. 47 was not significantly associated with the ES treatment (nonspecific). The cluster identifier number is shown on the x-axis, and -log p-values are indicated on the y-axis. The horizontal red dashed line shows a p-value of 0.05 by the binomial test (the probability of correlation was not significantly different from 50% for each of the clusters). (**C**) Hierarchical clustering revealed the similarity among cluster nos. 15, 23, 36 (ES-specific) and 47 (nonspecific) according to the overall behavioral profiles from the training set and test set. (**D**) Representative graph shows the counts of clusters 15, 23, and 36 (ES-specific) from the ES and QT phases.

We used the model to evaluate preidentified behavior features in new data as a demonstration. The data set was divided into a training set of 80% segments and a test set of 20% segments. The model was trained on the 5 s segment training set data by using the same cluster analysis condition as that used on the full data set (cumulative proportion of variance explained: 72% and vigilance for each cluster: 0.73), and the treatment-specific behavior features were identified. The test set was used to evaluate the behavior feature preidentified on the training set (Fig. 6A, Fig. S1). The behavior features of clusters 15, 23, and 36 were identified as ES-specific behavior features (p < 0.05, binomial test), and the behavior features of cluster 47 were identified as nonspecific behavior features (Fig. 6B). Next, we used the model fit by the training set data to evaluate the behavior features in test set data. The test set data included data from the ES phase and data from the QT phase: Hierarchical clustering revealed the similarities in the overall behavioral profiles among clusters 15, 23, 36 (ES-specific) and 71 (nonspecific) in the training set and test set (Fig. 6C). As shown in the figure, considerable differences were observed in the ES-specific behavior features and nonspecific behavior features based on the traditional behavioral quantitative analysis, which suggests that our system could efficiently classify swimming paths in 3D space. In addition, for the same number of cluster indexes, the behaviors of the training set and the test set showed a high degree of similarity in traditional quantitative analysis of behavior. Then, we also present a quantitative comparison of the count of each cluster from the ES phase and QT phase. Cluster nos. 15, 23, and 36 (ES-specific behavior features) were only found in the ES phase and not in the QT phase (Fig. 6D). These results showed that our system is suitable for the quantitative detection of behavior features that have been defined in advance using any data set.

## Discussion

Marques, João C., et al. analyzed the 2D behavior of zebrafish larvae by using unsupervised behavioral clustering^38^, and Hughes, G. L., et al. classified parkinsonian adult zebrafish using 2D behavior data and machine learning^39^; however, these authors did not analyze the 3D behavior features of zebrafish. Zebrafish exhibit complex 3D swimming patterns^15^, and MacRì et al. found that traditional behavioral scoring of individual zebrafish based on 2D analyses may lower the data integrity; thus, scoring zebrafish behavior by incorporating a 3D approach may be required^16^. Based on the above issues, this paper provides a solution to reconstructing 3D behavior data and reducing the multicamera time delay^31,34^. Previous studies have used machine learning to analyze behavior characteristics^38,39^; however, only 2D data were used. Considering the inaccuracy of 2D versus 3D, in the present study, we developed a FuzzyART program to mitigate these problems. In our previous study, FuzzyART was used to extract the common features of genetic networks using experimental time series microarray data^37^. Our study is the first to find that animal behavioral spatiotemporal features could also be classified by using the FuzzyART model. Moreover, for the new input data, we could detect preidentified treatment-specific behavior features by using a previously trained model. This result suggests that FuzzyART has high potential to function as a new method for obtaining animal behavior features based on machine learning fit models rather than traditional text or parameter descriptions, and then the duplicated model can be used in another context by different researchers to conveniently and effectively detect and quantitatively analyze newly predefined behavior features. Given that a large amount of animal behavior data can be captured, this method could help researchers configure various treatments and gene-edited lines and investigate and communicate the treatment-specific or mutation-specific behavioral features in small fish models.

## Conclusions

We developed a 3D swim path reconstruction system that was automatically controlled by an Arduino controlled using our developed program and presented a novel approach to classifying animal behavioral features based on 3D spatiotemporal data. Moreover, for the newly defined behavior pattern obtained by the 3D tracking analysis, we developed a tool to accept user-supplied data for the detection and quantitative analysis of behavioral features. This technique could be applied for the discovery of a new behavior patterns in mutant zebrafish and used for drug administration screening and cognitive ability tests of zebrafish in the future.

## Supplementary Information

Supplementary Video 1.

Supplementary files.
