# Supplementary material for: Zebrafish behavior feature recognition using three-dimensional tracking and machine learning
Source: Sci Rep. 2021 Jun 29;11:13492. doi: 10.1038/s41598-021-92854-0 (PMC8242018; doi:10.1038/s41598-021-92854-0)
Supplement: Supplementary file 2 — Supplementary files. [file 41598_2021_92854_MOESM2_ESM.docx]

Supplementary information

**
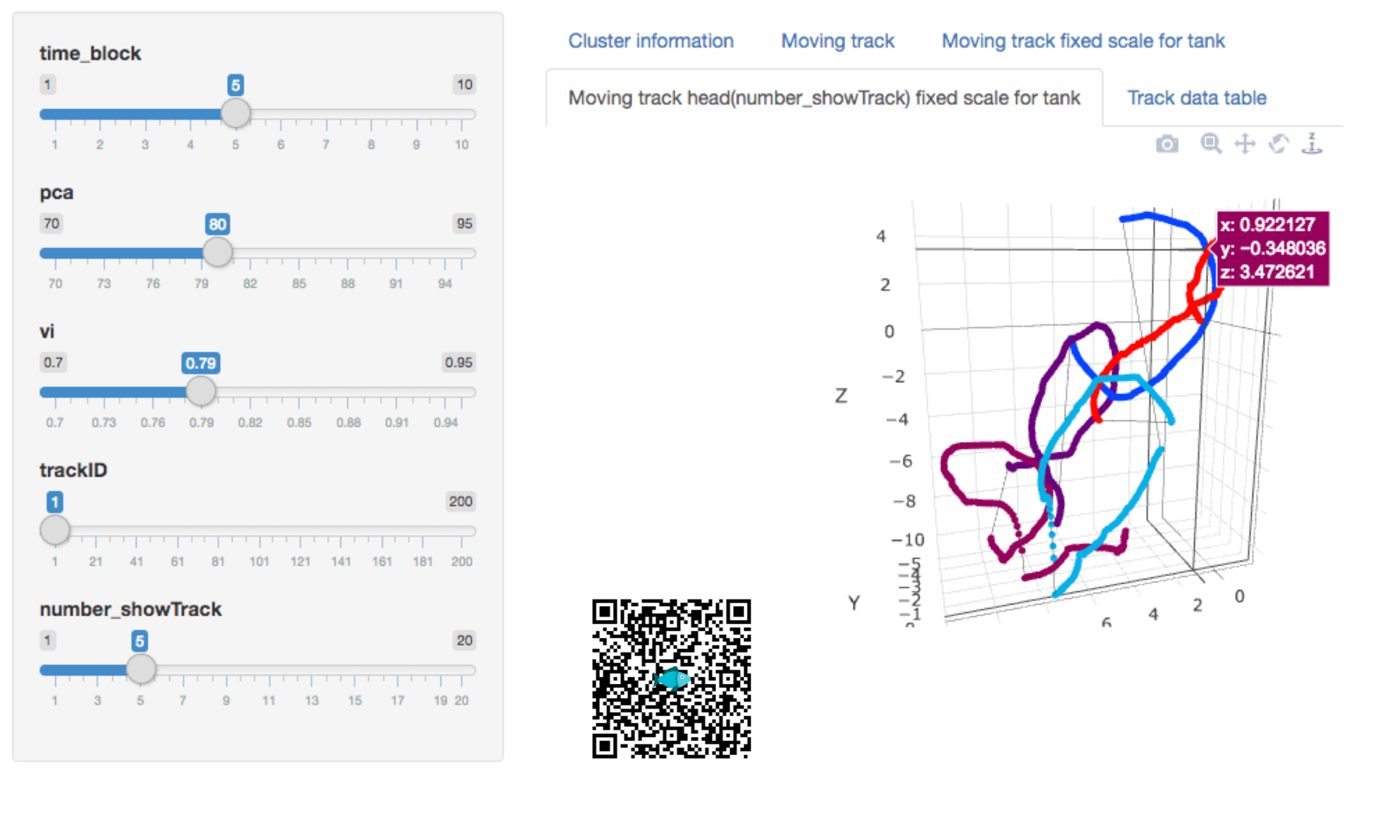
**

**Figure S1.** Interactive Web application “ShinyR-3D-zebrafish”. To decrease the complexity and time required to visualize and analyze the data, we developed a new, free, open-source, cloud-based application. Our program has an intuitive graphical user interface that enables novice users to quickly perform complex analyses. model parameter selection includes time segments (Time-block), the cumulative proportion of variance explained (pca), vigilance for each cluster (vi), Time when the behavior occurred (trackID), Number of behavior shown in the plot. Moreover, users also could check informative data tables, 3D-tracking plot or animation and Behavior 3D plot summary by this interactive web application “ShinyR-3D-zebrafish”

**Figure S2.** ES-specific behavior features screening in Fig.5. A scatter plot representation of shows the count from ES phase (x-axis) and Binomial test results significance (P-value, y-axis). Each dot represents a single cluster (behavior features). The horizontal red dash line shows a p-value of 0.05.

**Figure S3.** ES-specific behavior features screening of the training set in Fig.6. A scatter plot representation of shows the count from ES phase (x-axis) and Binomial test results significance (P-value, y-axis). Each dot represents a single cluster (behavior features). The horizontal red dash line shows a p-value of 0.05.

**Supplementary Video S1.** A video of 3D behavior features identified by machine learning.

The Video shows three groups of motion tracks of zebrafish identified by machine learning (Fig.5C, cluster 45, No.1, No.3, and No.5). Each group of motion tracks has two angles, which are taken by two cameras on the side and bottom.
